# VOLUNTEERING AND EPIGENETIC AGE AMONG RETIRED AND WORKING OLDER ADULTS: EVIDENCE FROM HEALTH AND RETIREMENT STUDY

**DOI:** 10.1093/geroni/igae098.0974

**Published:** 2024-12-31

**Authors:** Seoyoun Kim, Cal Halvorsen, Claire Potter

**Affiliations:** Texas State University, San Marcos, Texas, United States; Washington University in St. Louis, St. Louis, Missouri, United States; Queen’s University Belfast, Belfast, Northern Ireland, United Kingdom

## Abstract

Biological aging, distinct from chronological age, shows significant intra-individual variations particularly in later life. Volunteering, a modifiable lifestyle factor, correlated with improved physical health outcomes. Recent studies suggest a potential link between frequent volunteering and slower epigenetic aging, crucial in understanding biological aging mechanisms. However, changes in volunteering, selection biases, and life transitions impact this association. Using the 2014-2016 wave of Health and Retirement Study (HRS), including DNA methylation (DNAm) assays from the Venous Blood Study, we analyzed volunteering frequency (both contemporaneous and cumulative) and five DNAm clocks (Horvath, Hannum, PhenoAge, GrimAge, DunedinPoAm) in working and retired individuals. Each DNAm measure was regressed on chronological age and cell types and was standardized for ease of comparison. We also considered selection into volunteering in 2014 via Inverse Probability Treatment Weighting. Findings show that low to medium level volunteering (1-50 hrs per year) predicted slower aging for Horvath and Hannum clocks, while GrimAge and DunedinPoAm showed deceleration with higher level volunteering (100+ hrs per year). Further, higher cumulative volunteering index was related with slower epigenetic aging acceleration for all clocks. Stratification by retirement status revealed nuanced effects, with higher volunteering hours being more beneficial for workers in GrimAge and DunedinPoAm. These findings indicate volunteering’s net effects on epigenetic age among older adults. However, frequent volunteering might be more protective against epigenetic age acceleration among workers compared to retirees. Understanding these dynamic relationships offers insights for public health interventions to decelerate biological aging and extend healthy life expectancy.

